# Liposomal irinotecan (HR070803) in combination with 5-fluorouracil and leucovorin in patients with advanced solid tumors: a phase 1b dose-escalation and expansion study

**DOI:** 10.1007/s10637-024-01442-2

**Published:** 2024-07-22

**Authors:** Dongmei Ji, Weina Shen, Ting Li, Huan Wang, Jianling Bai, Junning Cao, Xichun Hu

**Affiliations:** 1 Department of Medical Oncology, Department of Oncology, Fudan University Shanghai Cancer Center, Fudan University, No. 273, Dongan Road, Shanghai, 200032 People’s Republic of China; 2 Department of Oncology, Shanghai Medical College, Fudan University, Shanghai, People’s Republic of China; 3 Jiangsu Hengrui Pharmaceuticals Co., Ltd, Shanghai, People’s Republic of China; 4 Department of Biostatistics, School of Public Heath, Nanjing Medical University, Nanjing, Jiangsu People’s Republic of China

**Keywords:** HR070803, Nanoliposomal irinotecan, Dose-limiting toxicity, Maximum tolerated dose, Pharmacokinetics, Advanced solid tumors

## Abstract

**Abstract:**

This phase 1b study aimed to evaluate the dose-limiting toxicity (DLT), maximum tolerated dose (MTD), pharmacokinetics, and preliminary efficacy of HR070803, a novel nanoliposomal formulation of irinotecan, in combination with 5-fluorouracil and leucovorin in patients with pretreated advanced solid tumors. This study consisted of dose-escalation and expansion stages. Dose escalation was performed with a traditional 3 + 3 design; patients received intravenous infusion of HR070803 from 60 to 80 mg/m^2^, followed by leucovorin (200 mg/m^2^) and 5-fluorouracil (2000 mg/m^2^) every 2 weeks. In the expansion stage, patients received treatments at selected tolerable dose. Fifteen patients received treatments at 60 mg/m^2^ (*n* = 12) and 80 mg/m^2^ (*n* = 3). DLTs occurred in 2 patients at 80 mg/m^2^ (grade 2 neutropenia that resulted in a dose delay of ≥ 7 days, *n* = 1; grade 3 febrile neutropenia, *n* = 1). The MTD was determined to be 60 mg/m^2^. The most frequent HR070803related adverse events included anorexia, leukopenia, neutropenia, nausea, fatigue, and diarrhea. SN-38, the active metabolite of irinotecan, exhibited lower maximum plasma concentrations and a prolonged terminal half-life when irinotecan was administered via nanoliposome compared to conventional injection. Overall, 4 patients achieved a partial response (confirmed, *n* = 2), and 9 had stable disease. The MTD of HR070803 was 60 mg/m^2^ when infused with 5-fluorouracil and leucovorin. Nanoliposomal encapsulation modified the pharmacokinetics of irinotecan and SN-38. HR070803 with 5-fluorouracil and leucovorin demonstrated a manageable safety profile and promising antitumor efficacy in advanced solid tumors.

**Trial registration:**

Clinicaltrials.gov, NCT05086848. Retrospectively registered on Oct. 12, 2021.

## Introduction

Since it was first approved in 1994, irinotecan (CPT-11) has been used to treat solid tumors worldwide as a monotherapy and, more frequently, in combination with other cytotoxic agents and monoclonal antibodies [1]. However, severe side effects, primarily neutropenia and late-onset diarrhea, often lead to the interruption and/or discontinuation of treatment, thus precluding a large therapeutic index and adversely affecting patient prognosis and quality of life [2]. Nanoliposomal formulations of irinotecan were subsequently developed to reduce drugrelated toxicity and enhance antitumor efficacy [3]. Nanoliposomal encapsulation contributed to a decrease in the maximum plasma concentration (C_max_), which alleviated doserelated side effects, and prolonged the plasma circulation time by preventing the rapid conversion of irinotecan [4]. In addition, nanoliposome carriers have the advantages of increased permeability and enhanced retention at the sites of solid tumors, indicating potential improvements in antitumor efficacy [5].

PEP02 was the first liposomal irinotecan developed by Ipsen Biopharmaceuticals and was approved by the US FDA for the treatment of metastatic pancreatic adenocarcinoma after progression following gemcitabine-based therapy when combined with 5-fluorouracil (5-FU) and leucovorin (LV). The approval of this formulation was primarily based on the pivotal phase 3 NAPOLI-1 study [6].

HR070803 is a novel liposomal irinotecan with a nanoliposome formulation and a preparation process that differs from that of PEP02. In preclinical studies, HR070803 demonstrated strong antitumor activity and a favorable toxicology profile in various nude mouse xenograft models owing to the longer halflife and lower C_max_ due to nanosized liposome encapsulation (undisclosed data). In our previous phase 1a study of HR070803 monotherapy at doses ranging from 80 to 160 mg/m^2^, dose-limiting toxicity (DLT) was observed in 2 patients at 160 mg/m^2^: one had grade 3 hyponatremia, and the other had grade 3 diarrhea. The maximum tolerated dose (MTD) was determined to be 120 mg/m^2^ at a 3-week interval (undisclosed data). Irinotecan hydrochloride injection (Camptosar®) in combination with 5-FU and LV has been approved as the first-line therapy for metastatic colon or rectal carcinoma [7, 8]. A synergistic effect was also observed with the sequential injection of irinotecan liposomes and 5-FU/LV [6, 9–11]. Based on these preclinical and clinical findings, it is reasonable to expect that the combination of HR070803 and 5-FU/LV has the potential to address an unmet need for the treatment of patients with advanced solid tumors.

Here, we report the results of an open-label, phase 1b, dose-escalation and expansion study that was conducted to assess the safety, tolerability, pharmacokinetic characteristics, and preliminary efficacy of HR070803 in combination with 5-FU and LV in patients with pretreated advanced solid tumors.

## Methods

### Patient selection

The key inclusion criteria included the following: (1) histologically and/or cytologically confirmed diagnosis of advanced solid tumor for which standard treatment failed or standard medical treatment was inapplicable; (2) age ≥ 18 years and life expectancy ≥ 3 months; (3) Eastern Cooperative Oncology Group performance status (ECOG PS) of 0 or 1; (4) adequate hematologic (absolute neutrophil count [ANC] ≥ 1.5 × 10^9^/L, platelet count ≥ 100 × 10^9^/L and hemoglobin ≥ 9 g/dL), hepatic (total bilirubin ≤ 1.5×upper limit of normal [ULN] and aspartate aminotransferase [AST]/alanine aminotransferase [ALT] ≤ 3×ULN [ALT/AST ≤ 5×ULN for patients with liver metastasis]), renal (creatinine clearance rate ≥ 60 mL/min calculated by the Cockcroft-Gault formula and serum creatinine ≤ 1.5×ULN), and cardiac (normal electrocardiograms with QTcB < 450 ms for males, QTcB < 470 ms for females) functions.

The key exclusion criteria were as follows: (1) known or suspicious primary or secondary brain tumors; (2) uridine-diphosphate glucuronosyltransferase 1A1 (UGT1A1)*28 homozygous 7/7 variant; (3) history of allergic reaction to liposomes; (4) significant cardiovascular diseases (including New York Heart Association ≥ level II); (5) severe gastrointestinal dysfunction; and (6) severe diseases related to safety or the completion of the trial confirmed by investigators.

Written informed consent forms were signed by all patients prior to initiating any studyrelated activities. The protocol and its amendments as well as the informed consent form were reviewed and approved by the independent ethics committee of Fudan University Shanghai Cancer Center (Shanghai, China). This study was registered at chinadrugtrials.org.cn (CTR20150626) before initiation and retrospectively registered at clinicaltrials.gov (NCT05086848). This trial conformed to the International Conference on Harmonization Good Clinical Practice guidelines and Good Clinical Laboratory Practice.

### Treatments and dose escalation strategy

This study was performed in 2 stages, namely, the dose-escalation stage and the expansion stage. In the dose-escalation stage, a traditional 3 + 3 design was utilized. The HR070803 dose started at 60 mg/m^2^ (calculated on the basis of anhydrous irinotecan hydrochloride), and it was escalated by 30% (80 mg/m^2^), 25% (100 mg/m^2^), and 20% (120 mg/m^2^) sequentially. Dose escalation was carried out if 3 patients successfully completed the DLT observation period (i.e., 28 days) and did not experience any DLT. If 1 of the 3 patients had DLT, 3 more patients were enrolled at this dose level. If ≥ 2 patients had DLTs, no more patients were enrolled at this dose level and dose escalation was stopped; the lower dose level was considered the MTD. Therefore, MTD was the highest dose at which no more than 1 patient experienced DLT. At each dose level, patients were sequentially intravenously infused with HR070803, LV (200 mg/m^2^), and 5-FU (2000 mg/m^2^) every 2 weeks (Q2W). Patients received study treatments until radiological progressive disease (PD) or unacceptable toxicity, as determined by the investigator.

In the dose expansion stage, additional patients were enrolled in the recommended dose group to more comprehensively evaluate the safety, tolerability, pharmacokinetic characteristics, and preliminary efficacy of the combined regimen.

### DLT definition and safety assessments

DLT was defined as the occurrence of at least one of the following events during the 28-day DLT observation period: (1) grade 3 or 4 nonhematological toxicities, except for nausea, vomiting, or diarrhea without treatment; (2) grade 4 neutropenia lasting for ≥ 5 days or grade ≥ 3 febrile neutropenia (ANC < 1000/mm^3^ together with a single oral temperature measurement of ≥ 38.3 °C or ≥ 38.0 °C for 1 h); (3) grade 3 thrombocytopenia (25 × 10^9^/L ≤ platelet count < 50 × 10^9^/L) with bleeding or grade 4 thrombocytopenia; (4) other grade 4 hematological toxicities; and (5) dose delay for more than 1 week owing to drug-related toxicity during the second or third treatment cycle.

All the patients underwent physical examinations and clinical laboratory assessments. Adverse events (AEs) were evaluated by investigators based on the National Cancer Institute (NCI) Common Terminology Criteria for Adverse Events (CTCAE) v4.03. The causal relationships between AEs and the study treatments were assessed by the investigator.

### Pharmacokinetic analyses

Blood samples (4 mL) were collected from each patient 45 min before treatment, immediately at the end of infusion, and at 0.25, 0.5, 1, 1.5, 2, 3, 5, 8, 12, 24, 36, 48, 72, 96, 120, and 168 h after infusion. The plasma concentrations of total irinotecan, the active lactone and inactive carboxylate forms of free irinotecan (abbreviated as CPT-11_LAC and CPT-11_CAR, respectively), and the active lactone and inactive carboxylate forms of SN-38 (abbreviated as SN-38_LAC and SN-38_CAR, respectively) were quantified using liquid chromatography tandem mass spectrometry with a validated methodology. The plasma concentration versus time profiles were plotted. A noncompartment model (Phoenix WinNonlin version 7.0, Pharsight Corporation, USA) was used to calculate pharmacokinetic parameters, including the area under the concentration-time curve (AUC_0 − t_ and AUC_0−∞_), C_max_, time to C_max_ (T_max_), terminal half-life (t_1/2_), clearance (CL), and volume of distribution (V_ss_).

### Efficacy assessments

Assessments of response to treatments were performed every 3 cycles (6 weeks) based on Response Evaluation Criteria in Solid Tumors (RECIST) v1.1. If a complete response (CR) or partial response (PR) was documented, further confirmation was required 4 weeks later.

### Statistical analyses

Descriptive statistics were used in the analysis. Continuous variables are shown as the mean ± standard deviation (SD) or the median with the minimum and maximum; categorical variables are shown as frequencies. All the statistical analyses were performed using SAS version 9.4.

## Results

### Patient characteristics

This study was performed between May 5, 2016, and June 6, 2018, at a single center in China. A total of 15 patients were treated and evaluated for safety, pharmacokinetics, and efficacy. All the patients completed the DLT observation period.

Table 1 presents the demographics and baseline disease characteristics of the patients. The median age of all patients was 49.0 years, with a range of 21 to 70 years. There were 8 men (53.3%) and 7 women (46.7%). The most common tumor types were colorectal cancer (20.0%), nasopharyngeal cancer (20.0%), and breast cancer (13.3%). Fourteen patients (93.3%) had previously received chemotherapy.

**Table 1:** Demographics and baseline disease characteristics

Characteristics	60 mg/m^2^ (*n* = 12)	80 mg/m^2^ (*n* = 3)	Total (*n* = 15)
*Age (years)*			
Mean ± SD	46.8 ± 14.5	44.7 ± 21.8	46.4 ± 15.3
Median (range)	48.5 (26–70)	49.0 (21–64)	49.0 (21–70)
*Sex, n (%)*			
Male	5 (41.7)	3 (100)	8 (53.3)
Female	7 (58.3)	0 (0.0)	7 (46.7)
*ECOG PS, n (%)*			
0	0 (0.0)	0 (0.0)	0 (0.0)
1	12 (100)	3 (100)	15 (100)
*Tumor type, n (%)*			
Colorectal cancer	3 (25.0)	0 (0.0)	3 (20.0)
Breast cancer	2 (16.7)	0 (0.0)	2 (13.3)
Nasopharyngeal cancer	1 (8.3)	2 (66.7)	3 (20.0)
Periampullary carcinoma	1 (8.3)	0 (0.0)	1 (6.7)
Esophageal cancer	1 (8.3)	0 (0.0)	1 (6.7)
Laryngeal cancer	1 (8.3)	0 (0.0)	1 (6.7)
Pancreatic cancer	0 (0.0)	1 (33.3)	1 (6.7)
Others	3 (25.0)	0 (0.0)	3 (20.0)
*History of cancer treatment, n (%)*			
Surgery	10 (83.3)	1 (33.3)	11 (73.3)
Chemotherapy	11 (91.7)	3 (100)	14 (93.3)
Radiotherapy	7 (58.3)	1 (33.3)	8 (53.3)

ECOG PS, Eastern Cooperative Oncology Group performance status; N, number of patients; SD, standard deviation

### Dose escalation and MTD

No DLTs occurred at the starting dose of 60 mg/m^2^, and the dose was therefore further increased to 80 mg/m^2^, at which point two of the 3 patients experienced DLTs (Table 2). Specifically, 1 patient with nasopharyngeal carcinoma had grade 2 neutropenia that led to a dose delay of more than 7 days. This event occurred 14 days after the first administration, and the patient recovered after 8 days. Another patient with pancreatic cancer experienced grade 3 febrile neutropenia. This event was observed 13 days after the first administration. The symptoms disappeared after 2 days. The MTD of HR070803 was determined to be 60 mg/m^2^ when administered with 5-FU/LV. In the expansion stage, an additional 9 patients were enrolled in the 60 mg/m^2^ group, and no DLTs were observed.

**Table 2:** Dose escalation design

HR070803 dose	Proportion of dose increment	Number of patients treated	Number of patients with DLT
60 mg/m^2^	Starting dose	12^a^	0
80 mg/m^2^	33%	3	2

^a^ Three patients were enrolled in the dose-escalation stage, and 9 patients were enrolled in the expansion stage

### Safety and tolerability

All 15 patients had received ≥ 3 cycles of treatments and experienced at least 1 treatment-emergent adverse event (TEAE). The most frequently reported TEAEs included anorexia (86.7%), neutropenia (80.0%), leukopenia (80.0%), nausea (73.3%), diarrhea (60.0%), and fatigue (60.0%). Grade ≥ 3 TEAEs occurred in 10 patients (66.7%), and the most common events were neutropenia (33.3%), elevated conjugated bilirubin (13.3%), infection (13.3%), and diarrhea (13.3%). All the patients had experienced at least 1 HR070803-related AE (Table 3), with the most common being anorexia (86.7%), leukopenia (80.0%), neutropenia (80.0%), nausea (73.3%), fatigue (60.0%), and diarrhea (60.0%). Nine (60.0%) patients had HR070803-related AEs of grade ≥ 3, and the most commonly reported were neutropenia (33.3%), elevated conjugated bilirubin (13.3%), and diarrhea (13.3%). Three patients experienced serious adverse events (SAEs), including one case each of septicemia (HR070803 60 mg/m^2^), infection (HR070803 60 mg/m^2^), and febrile neutropenia (HR070803 80 mg/m^2^); the infection case was deemed unrelated to HR070803. No deaths occurred in this study. No patients discontinued treatments due to TEAEs.

**Table 3:** HR070803-related adverse events

Preferred term (*n*, %)	Total (*n* = 15)		60 mg/m^2^ (*n* = 12)		80 mg/m^2^ (*n* = 3)	
	Any grade	Grade ≥ 3	Any grade	Grade ≥ 3	Any grade	Grade ≥ 3
Total	15 (100)	9 (60.0)	12 (100)	8 (66.7)	3 (100)	1 (33.3)
Anorexia	13 (86.7)	1 (6.7)	10 (83.3)	1 (8.3)	3 (100)	0 (0)
Leukopenia	12 (80.0)	1 (6.7)	10 (83.3)	1 (8.3)	2 (66.7)	0 (0)
Neutropenia	12 (80.0)	5 (33.3)	10 (83.3)	4 (33.3)	2 (66.7)	1 (33.3)
Nausea	11 (73.3)	0 (0)	9 (75.0)	0 (0)	2 (66.7)	0 (0)
Fatigue	9 (60.0)	1 (6.7)	7 (58.3)	1 (8.3)	2 (66.7)	0 (0)
Diarrhea	9 (60.0)	2 (13.3)	7 (58.3)	2 (16.7)	2 (66.7)	0 (0)
Decreased body weight	6 (40.0)	0 (0)	5 (41.7)	0 (0)	1 (33.3)	0 (0)
Elevated conjugated bilirubin	5 (33.3)	2 (13.3)	4 (33.3)	1 (8.3)	1 (33.3)	1 (33.3)
Anemia	5 (33.3)	0 (0)	4 (33.3)	0 (0)	1 (33.3)	0 (0)
Vomiting	4 (26.7)	0 (0)	3 (25.0)	0 (0)	1 (33.3)	0 (0)
Albuminuria	4 (26.7)	0 (0)	3 (25.0)	0 (0)	1 (33.3)	0 (0)
Oral mucositis	4 (26.7)	1 (6.7)	4 (33.3)	1 (8.3)	0 (0)	0 (0)
Increased ALT	3 (20.0)	0 (0)	2 (16.7)	0 (0)	1 (33.3)	0 (0)
Increased AST	3 (20.0)	0 (0)	2 (16.7)	0 (0)	1 (33.3)	0 (0)
Elevated blood bilirubin	3 (20.0)	0 (0)	2 (16.7)	0 (0)	1 (33.3)	0 (0)
Feeble	3 (20.0)	1 (6.7)	3 (25.0)	1 (8.3)	0 (0)	0 (0)
Abdominal pain	3 (20.0)	0 (0)	3 (25.0)	0 (0)	0 (0)	0 (0)
Alopecia	3 (20.0)	0 (0)	3 (25.0)	0 (0)	0 (0)	0 (0)
Chest discomfort	2 (13.3)	1 (6.7)	2 (16.7)	1 (8.3)	0 (0)	0 (0)
Dizziness	2 (13.3)	0 (0)	2 (16.7)	0 (0)	0 (0)	0 (0)
Hyponatremia	2 (13.3)	1 (6.7)	1 (8.3)	0 (0)	1 (33.3)	1 (33.3)
Pyohemia	1 (6.7)	1 (6.7)	1 (8.3)	1 (8.3)	0 (0)	0 (0)
Febrile neutropenia	1 (6.7)	1 (6.7)	0 (0)	0 (0)	1 (33.3)	1 (33.3)

HR070803-related AEs with an incidence of ≥ 10% in either group and all events of grade ≥ 3 are included in the table

ALT, alanine aminotransferase; AST, aspartate aminotransferase

### Pharmacokinetics

**Fig. 1:**
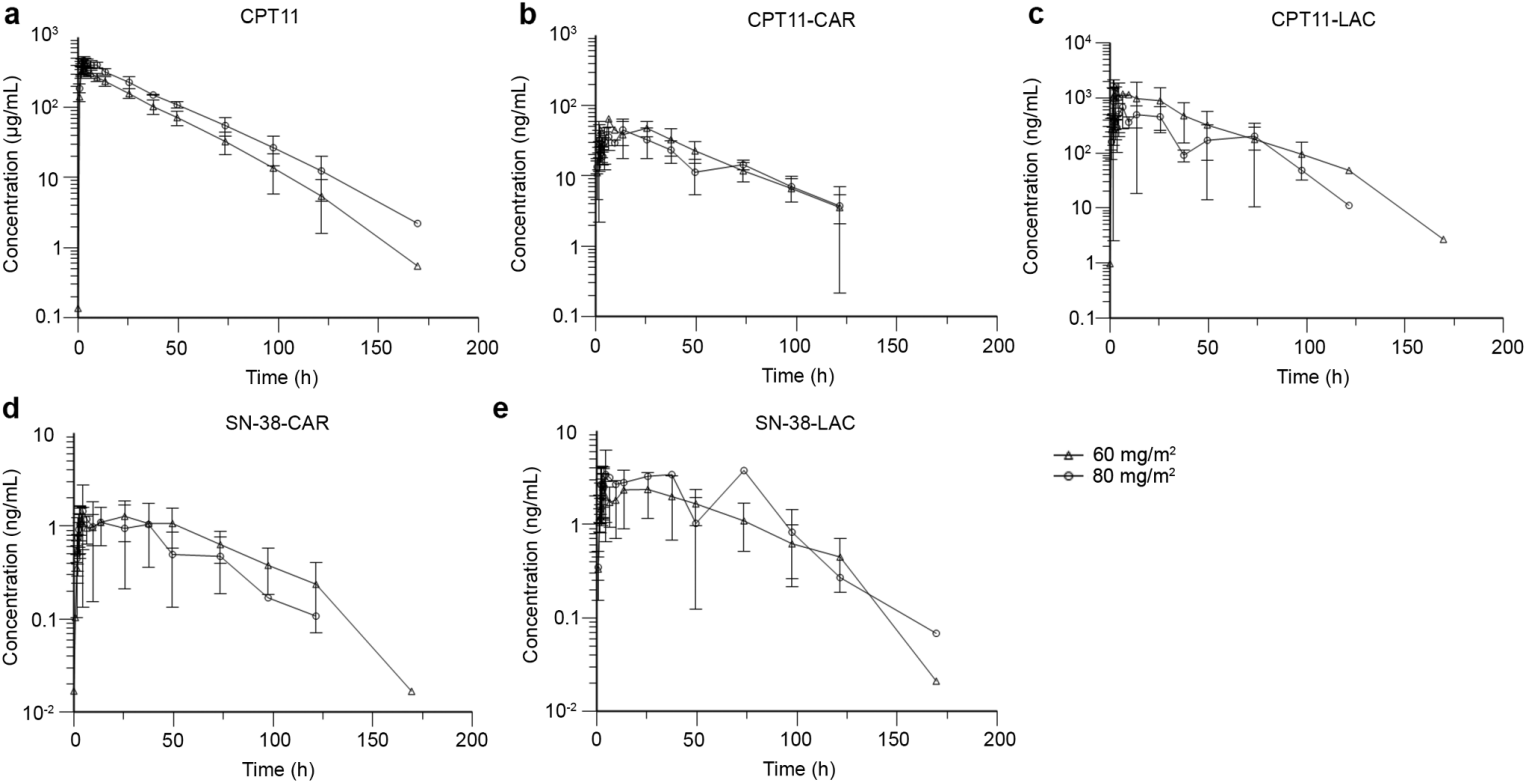
Mean plasma concentration-time curve of irinotecan and SN-38 after administration of HR070803. (**a**) Total irinotecan; (**b**) Inactive carboxylate form of irinotecan; (**c**) Active lactone form of irinotecan; (**d**) Inactive carboxylate form of SN-38; (**e**) Active lactone form of SN-38. Data are mean (standard deviation)

The mean plasma concentrations-time curves of total irinotecan, CPT-11_LAC, CPT-11_CAR, SN-38_LAC, and SN-38_CAR are shown in Fig. 1. The pharmacokinetic parameters of total irinotecan, CPT-11_LAC, CPT-11_CAR, SN-38_LAC, and SN-38_CAR are summarized in Table 4. For total irinotecan and SN-38_LAC, both the C_max_ and AUC_0 − t_ increased with dose over the range of 60 to 80 mg/m^2^, whereas for CPT-11_LAC, CPT-11_CAR and SN-38_CAR, such a trend was not observed. This might be due to the small number of patients, especially in the 80 mg/m^2^ group (*N* = 3), and the interpatient variability. It was also difficult to determine the doseproportionality of the pharmacokinetics of different forms of irinotecan and SN-38. At a dose of 60 mg/m^2^, the mean AUC_0 − t_ of CPT-11_LAC was approximately 19-fold greater than that of CPT-11_CAR; the mean AUC_0 − t_ of SN-38_LAC was approximately twice that of SN-38_CAR. The CL (0.0533 in the 60 mg/m^2^ group vs. 0.0471 L/h/m^2^ in the 80 mg/m^2^ group) and V_ss_ (1.52 L/m^2^ for both groups) of total irinotecan were comparable between the 2 dose groups.

**Table 4:** Pharmacokinetic parameters of irinotecan and its metabolites in HR070803

Parent compounds	t_1/2_ (h)	T_max_ (h)	C_max_ (µg/mL)	AUC_0 − t_ (h·µg/mL)	AUC_0−∞_ (h·µg/mL)
*Total irinotecan*					
60 mg/m^2^ (*n* = 12)	18.2 (3.12)	3.25 (2.00-6.50)	34.4 (3.91)	1080 (180)	1090 (181)
80 mg/m^2^ (*n* = 3)	21.1 (2.07)	2.00 (1.75-3.00)	47.7 (5.55)	1600 (164)	1610 (160)
*CPT-11_LAC*					
60 mg/m^2^ (*n* = 12)	26.4 (11.0)	4.00 (1.50–25.5)	2.94 (2.16)	49.1 (22.7)	51.4 (24.0)
80 mg/m^2^ (*n* = 3)	17.4 (5.66)	6.50 (2.00-6.50)	1.14 (0.384)	24.6 (3.66)	25.1 (3.88)
*CPT-11_CAR*					
60 mg/m^2^ (*n* = 12)	28.1 (9.38)	6.50 (1.50–25.5)	0.117 (0.109)	2.61 (0.714)	2.84 (0.766)
80 mg/m^2^ (*n* = 3)	31.2 (7.03)	4.50 (2.00-13.5)	0.0572 (0.0079)	2.11 (0.227)	2.29 (0.187)
*Metabolites*					
*SN-38_LAC*					
60 mg/m^2^ (*n* = 12)	37.0 (13.1)	3.50 (3.00-4.50)	3.22 (1.34)	168 (68.5)	191 (72.0)
80 mg/m^2^ (*n* = 3)	56.0 (10.2)	73.5 (2.00-73.5)	4.68 (3.89)	265 (299)	118 (11.5)
*SN-38_CAR*					
60 mg/m^2^ (*n* = 12)	44.7 (18.4)	5.50 (3.50–73.5)	1.64 (0.621)	92.7 (32.1)	113 (30.6)
80 mg/m^2^ (*n* = 3)	65.4 (26.7)	11.5 (3.00-49.5)	1.75 (1.05)	66.7 (65.4)	97.2 (56.1)

The mean (SD) is provided for each pharmacokinetic parameter, except for T_max_, for which the median (range) is presented

AUC, area under the concentration‒time curve; C_max_, maximum plasma concentration; CPT-11_CAR, carboxylate form of free irinotecan; CPT-11_LAC, lactone form of free irinotecan; N, number of patients; SD, standard deviation; SN-38_CAR, carboxylate form of SN-38; SN-38_LAC, lactone form of SN-38; t_1/2_, elimination halflife; T_max_, time to C_max_

### Efficacy

**Fig. 2:**
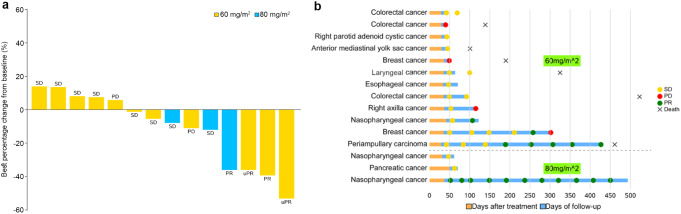
Response to treatments. (**a**) Waterfall plot of the best percentage change from baseline in the target lesions. (**b**) Response to treatments over time. One patient in the 60 mg/m^2^ group had only nontarget lesions; therefore, data on lesion changes were unavailable. PD, progressive disease; PR, partial response; SD, stable disease; uPR, unconfirmed partial response

Overall, two patients (13.3%) achieved confirmed PR, 2 patients (13.3%) achieved unconfirmed PR, and 9 patients (60.0%) exhibited stable disease (Fig. 2). A confirmed PR was observed in 1 patient with nasopharyngeal carcinoma at the dose of 80 mg/m^2^, and the duration of response (DoR) was 12.9 months. Another confirmed PR was observed in a patient with ampullary carcinoma in the 60 mg/m^2^ group, with a DoR of 7.9 months. Unconfirmed PR was observed in 1 patient each with nasopharyngeal carcinoma and breast cancer at the dose of 60 mg/m^2^. At the MTD (60 mg/m^2^), 1 patient had confirmed PR, 2 patients had unconfirmed PR, and 7 patients had stable disease. The objective response rate (ORR) was 13.3% (95% confidence interval [CI]: 1.7, 40.5), and the disease control rate (DCR) was 86.7% (95% CI: 59.5, 98.3).

## Discussion

In this phase 1b trial, the safety profile, pharmacokinetic characteristics, and preliminary efficacy of HR070803 combined with 5-FU and LV were assessed in patients with advanced solid tumors. DLTs that occurred were associated with myelosuppression, and the MTD of HR070803, which is also the recommended phase 2 dose, was determined to be 60 mg/m^2^. The pharmacokinetic results indicated that compared with conventional nonliposomal irinotecan injection, the C_max_ of SN-38 in HR070803 was lower, and the t_1/2_ was longer. In terms of treatment efficacy, 2 patients had confirmed PR, 2 patients had unconfirmed PR, and 9 patients had SD.

Because irinotecan damages blood cells, epithelial cells, and commensal bacteria, treatment with irinotecan is commonly accompanied by hematologic and gastrointestinal toxicities [1]. In our study, both DLTs observed were myelosuppressive (neutropenia and febrile neutropenia), and no gastrointestinal DLTs occurred; this result might be attributed to the small sample size and interpatient variations. A phase 1 study assessed the MTD, pharmacokinetics and preliminary efficacy of PEP02 in combination with 5-FU and LV when administered every 3 weeks in patients with advanced solid tumors [12]. The DLTs were myelosuppression and gastrointestinal events, including diarrhea, neutropenia, infection with hypotension, and hemorrhage. Another phase 1 study evaluated the MTD, pharmacokinetics, and preliminary efficacy of irinotecan liposome injection (LY01610) when administered Q2W in patients with advanced esophageal squamous cell carcinoma [10]. The DLTs observed were vomiting and febrile neutropenia [10]. The DLT results from the abovementioned phase 1 MTD exploration studies of liposomal irinotecan indicated that nanoliposome encapsulation did not impact the safety profile of irinotecan and, more importantly, did not cause new safety concerns.

In recent years, several phase 1 studies of liposomal irinotecan, used either as monotherapy or in combination with other therapeutic agents, have been conducted in patients with various tumors, such as metastatic breast cancer [13], advanced esophageal squamous cell carcinoma [10], recurrent high-grade glioma [9], pancreatic ductal adenocarcinoma [14], and advanced refractory solid tumors [4, 12]. The MTDs of different liposomal irinotecan formulations in these studies ranged from 50 mg/m^2^ to 120 mg/m^2^, whereas it was determined to be 60 mg/m^2^ in this study. The disparities in MTD might be attributed to different treatments, administration schedules, tumor types, and nanoliposome formulations as well as interpatient variation.

No new safety signals were identified with HR070803 compared with conventional irinotecan injection [7, 8]. Most TEAEs were of grade 1 or 2 severity, and they were resolved after appropriate supportive therapies or dose interruptions. No patients discontinued treatment due to TEAEs, and no deaths occurred. Cholinergic reactions can occur upon injection of irinotecan [7, 8, 15, 16]. However, in this study, no cholinergic syndrome was observed. The manageable and tolerable safety profile of HR070803 plus 5-FU/LV renders it a feasible treatment modality for patients with advanced solid tumors.

The pharmacokinetic parameters of HR070803 monotherapy at 80 mg/m^2^ in our previous phase 1a study (undisclosed data) and HR070803 plus 5-FU/LV at 80 mg/m^2^ in this study were similar, suggesting that 5-FU and LV exerted weak effects on the pharmacokinetic profile of HR070803. Compared with a previous study that assessed plasma SN-38 concentrations after the injection of nonliposomal irinotecan at 180 mg/m^2^ (Q2W) [17], the mean C_max_ of SN-38_LAC after administration of HR070803 at the MTD (60 mg/m^2^) was much lower (3.22 ng/mL vs. 26.2 ng/mL), as was its mean AUC_0∍∞_ (191 h·ng/mL vs. 368 h·ng/mL). The t_1/2_ of SN-38_LAC was longer after HR070803 administration compared with that after conventional irinotecan injection (mean value: 37.0 h vs. 19.7 h). Both irinotecan and its metabolite SN-38 exist in equilibrium between an active lactone form and an inactive carboxylate form, depending on the pH and binding proteins, after intravenous injection [4, 12, 18]. An acidic pH, such as that found in the tumor microenvironment, promotes the formation of the active lactone form. A liposome-encapsulated formulation could theoretically shift the equilibrium, resulting in greater amounts of the active lactone form within tumor tissue, enhancing the activity of irinotecan [5]. In this study, in addition to total irinotecan and active SN-38_LAC, we also assessed the plasma concentrations and AUCs of CPT-11_LAC, CPT-11_CAR, and SN-38_CAR, which made it possible to perform comparisons between the active LAC form and the inactive CAR form of irinotecan and SN-38. At the MTD (60 mg/m^2^), the mean AUC_0 − t_ of CPT-11_LAC was approximately 19fold greater than that of CPT-11_CAR. The mean ratio of lactone to total free CPT-11 was > 90% after HR070803 administration, which compared favorably with the ratio of 34-44% observed after the administration of the nonliposome formulations [18, 19]. The mean AUC_0 − t_ of SN-38_LAC was approximately 2-fold greater than that of SN-38_CAR. These results were consistent with previous findings showing that the lactone form of SN-38 accounted for approximately 60–70% of the total plasma SN-38 levels after the intravenous injection of irinotecan [18].

The active metabolite of irinotecan, SN-38, is inactivated and detoxified by the UGT enzyme, which is encoded by the *UGT1A1* gene [20]. Reduced UGT enzyme activity caused by genetic variation, such as the homozygous UGT1A1*28 allele, leads to decreased detoxification and persistence of SN-38, thus resulting in increased toxicity [20]. Considering that this study aimed to determine the MTD of HR070803 in combination with 5-FU/LV, patients with homozygous UGT1A1*28 were excluded. There were 5 patients who were heterozygous for UGT1A1*28 in this study. We also assessed the UGT1A1*6 variation in the patients. There was 1 patient with homozygous UGT1A1*6 who experienced febrile neutropenia and 3 patients with heterozygous UGT1A1*6. No patients had concurrent mutations in UGT1A1*6 and UGT1A1*28. In patients with UGT1A1*6/28 mutations, the safety and pharmacokinetics results were similar to those in patients who expressed the wild-type gene. However, owing to the small sample size, these results should be interpreted with caution. Future PK/pharmacodynamic studies are warranted to draw firm conclusions on the correlations between UGT1A1 gene polymorphism and the tolerability and pharmacokinetics profiles of HR070803.

Although the interpretation of the efficacy results was limited by the small sample size and heterogeneous tumor types, the ORR (13.3%) and DCR (73.3%) results indicated the potential antitumor activity of HR070803 in combination with 5-FU/LV. Indeed, a randomized, double-blinded, parallel-controlled, phase 3 study (NCT05074589) was conducted to evaluate the efficacy and safety of HR070803 plus 5-FU/LV versus placebo plus 5-FU/LV as the second-line therapy for gemcitabinerefractory locally advanced or metastatic pancreatic cancer. Treatment with HR070803 plus 5-FU/LV resulted in statistically significant and clinically meaningful prolonged OS versus placebo plus 5-FU/LV, and the safety profile was manageable [21].

In conclusion, the DLTs observed in this study were associated with myelosuppression. The MTD of HR070803 was determined to be 60 mg/m^2^ (Q2W) when it was combined with 5-FU and LV, and this is also the recommended dose for future studies. HR070803 in combination with 5-FU and LV had a manageable safety profile and promising antitumor activity in patients with advanced solid tumors. Compared with conventional nonliposomal irinotecan, HR070803 administration resulted in a lower C_max_ and prolonged t_1/2_ of SN-38 owing to nanosized liposome encapsulation.

## Data Availability

All the data supporting the conclusions of this article are included in the text.

## **Abbreviations**

5-FU: 5-fluorouracil
AE: Adverse event
ALT: Alanine aminotransferase
ANC: Absolute neutrophil count
AST: Aspartate aminotransaminase
AUC: Area under the concentration-time curve
CTCAE: Common Terminology Criteria for Adverse Events
CI: Confidence interval
CL: Clearance
C_max_: Maximum plasma concentration
CPT-11_CAR: Carboxylate form of free irinotecan
CPT-11_LAC: Lactone form of free irinotecan
CR: Complete response
DCR: Disease control rate
DLT: Dose-limiting toxicity
DOR: Duration of response
ECG: Electrocardiogram
ECOG PS: Eastern Cooperative Oncology Group performance status
LV: Leucovorin
MTD: Maximum tolerated dose
NCI: National Cancer Institute
ORR: Objective response rate
PD: Progressive disease
PR: Partial response
Q2W: Every two weeks
RECIST: Response evaluation criteria in solid tumors
SAE: Serious adverse event
SD: Standard deviation
SN-38_CAR: Carboxylate form of SN-38
SN-38_LAC: Lactone form of SN-38
t_1/2_: Terminal half-life
TEAE: Treatment-emergent adverse event
T_max_: Time for C_max_
UGT1A1: Uridine-diphosphate glucuronosyltransferase 1A1
ULN: Upper limit of normal
V_ss_: Volume of distribution
